# Platelets Rich Plasma (PRP) Procedure in the Healing of Atonic Wounds

**DOI:** 10.3390/jcm12123890

**Published:** 2023-06-07

**Authors:** Maur Sebastian Horgos, Ovidiu Laurean Pop, Mircea Sandor, Ioan Lucian Borza, Rodica Anamaria Negrean, Adrian Cote, Andreea-Adriana Neamtu, Carmen Grierosu, Liliana Sachelarie, Anca Huniadi

**Affiliations:** 1 Department of Surgical Disciplines, Faculty of Medicine and Pharmacy, University of Oradea, 1st December Square No. 10, 410073 Oradea, Romania; mauhorgos@yahoo.com (M.S.H.); adrian.cote@gmail.com (A.C.); ancahuniadi@gmail.com (A.H.); 2 Department of Pathology, County Clinical Emergency Hospital, Faculty of Medicine and Pharmacy, University of Oradea, 1st December Square No. 10, 410087 Oradea, Romania; drovipop@yahoo.com; 3 Department of Morphological Disciplines, Faculty of Medicine and Pharmacy, University of Oradea, 1st December Square No. 10, 410073 Oradea, Romania; borzaioanlucian@yahoo.com; 4 Department of Preclinical Disciplines, Faculty of Medicine and Pharmacy, University of Oradea, 1st December Square No. 10, 410073 Oradea, Romania; rodicanegrean@yahoo.com; 5 Department of Surgical Disciplines, Developmental Biology Biochemistry & Molecular Biology Area Studies Chemistry Communication, Faculty of Medicine and Pharmacy & Dental Medicine, Vasile Goldis Western University, 310045 Arad, Romania; aneamtu94@gmail.com; 6 Department of Preclinical Disciplines, Faculty of Medicine, Apollonia University, Păcurari Street 11, 700511 Iași, Romania; grierosucarmen@yahoo.com

**Keywords:** atonic wound, new therapies, platelet rich plasma, evolution, healing

## Abstract

(1) Background: Patients suffering from chronic wounds report physical, mental, and social consequences due to their existence and care. There is a global need for tissue repair strategies and, in our case, for chronic wound healing. PRP therapy is based on the fact that platelet-derived growth factors (PGF) support the three phases of the wound healing and repair cascade (inflammation, proliferation, and remodeling); (2) Methods: A comparative study was carried out on two groups of patients with atonic wounds totaling a total of 80 cases as follows: a study group in which the PRP procedure was applied and a control group in which the biological product was not injected. The study was carried out in the surgery clinic of the Clinical Hospital C.F. Oradea City; (3) Results: A much faster healing was achieved in the case of patients who benefited from the platelet-rich plasma injection therapy compared to the group of patients in whom this therapy was not used. Three weeks after the plasma injection, a considerable reduction of the wound was evident, with some of the patients presenting with a closed wound; (4) Conclusions: The effect of PRP on the healing of chronic wounds is promising in most cases. A positive effect was also highlighted in terms of reducing treatment costs by considerably reducing the materials used as well as the number of hospitalizations for the same pathology.

## 1. Introduction

Chronic wounds have been defined as the result of a deviation in one of the physiological processes of progressive wound healing without restoring normal anatomical and functional appearance [1]. Chronic wounds are frequently complicated by impediments in the healing process, such as ischemia, necrotic tissue, bacterial loads, or high levels of pro-inflammatory matrix metalloproteinases [2]. Wound healing is a complex, dynamic process supported by a multitude of cellular events that must be closely coordinated to effectively repair damaged tissue.

Despite the considerable innate reparative capacity, the multiple cellular aspects of the body’s response in the healing processes of an individual can become attenuated, compromising the closure of wounds. This attenuation is most often the result of systemic pathological changes, such as those associated with old age or treatments applied both at the hospital level and at home. Unfortunately, these chronic wounds (primarily venous ulcers, bedsores, and diabetic foot ulcers) are a major area of research into new methods of treatment, increasing significantly on a global scale [3,4]. Essentially, the skin acts as a primary defense barrier, preventing drying out and mechanical, chemical, and thermal damage to internal structures [5]. Wound repair is classically simplified into four main phases: hemostasis, inflammation, proliferation, and dermal remodeling, which result in architectural and physiological restoration following injuries. However, with the use of modern and advanced technology for faster wound healing, the cost can be reduced substantially [6,7].

Emerging autologous cell therapies using platelet-rich plasma (PRP) applications have the potential to play adjuvant roles in a variety of treatment plans regarding regenerative medicine. Many different formulations of PRPs have been evaluated, which come from human, in vitro, and animal studies. However, recommendations from in vitro and animal research often lead to different clinical outcomes, as it is difficult to translate the results of non-clinical studies and methodological recommendations into human clinical treatment protocols [8,9,10,11,12].

The purpose of this research is to evidence the healing process through the application of the PRP procedure in wounds following the acceleration of the healing phases through the local injection of the obtained biological substrate, the PRP solution; as a result, various cascades are initiated in the healing process, contributing to site immuno-modulation, inflammatory processes, and angiogenesis to stimulate tissue healing and repair.

## 2. Materials and Methods

The study was performed for 14 months, between 1 October 2021–31 December 2022, and conducted in the Surgery Clinic of the CF Clinical Hospital Oradea, summing up a total of 80 patients hospitalized with pathologies with which the presence of atonic wounds was associated. The total number of patients was divided into 2 groups; the study group included 50 patients to whom the PRP procedure was applied, and the control group included 30 patients to whom this procedure was not performed.

The inclusion criteria were the age between 40–70 years of both genders, patients in whom other new therapies in wound healing have not been previously applied, and the presence of pathologies such as diabetes mellitus, chronic obliterating arteriopathy, chronic venous insufficiency that associates varicose ulcer, pathologies in which the wounds have a long evolution and negatively influence the daily routine of the patients and who signed the agreement to participate in the study.

The exclusion criteria include patients whose age is not in the range of 40–70 years, superinfection at the level of the wound (culture from the negative wound), the presence of chronic wounds of a traumatic/contusion nature, as well as other conditions that prevent participation in the study such as limited cooperation, serious diseases that intervene on living conditions (cancer, advanced renal failure, advanced liver failure, and other immunodepressed diseases—HIV, AIDS) or those who did not sign the participation agreement in the study. This study was approved by the local ethics committee of the institution in which it was carried out, in compliance with the ethical conditions according to the Helsinki Declaration.

On the day of admission, the patients were given a questionnaire for the subjective evaluation of the symptomatology and to be able to observe the possible impact of this therapy on the symptoms. The questionnaire was completed on day 0, the day of admission, and on days 5, 7, and 10 of the treatment. The intensity of the pain, the discomfort when walking, mobility, and the nictemeral discomfort, as well as the general condition related to the day of admission, were highlighted. In this questionnaire, numerical scales were introduced to highlight, as well as possible, the evolution and impact of the PRP procedure on the patient.

The treatment of the patients participating in the control group included both drug treatment with anti-inflammatories, pain relievers and vitamins and local treatment involving a rigorous daily toilet with various antiseptic compounds, except for PRP injection therapy. Previously, a biopsy was taken from the wound both in the patients in the control group and in the patients in the study group after an excisional debridement of the pathological necrotic or fibrinous tissue where appropriate. The biopsies were performed on the day of admission, respectively 21 days after the PRP procedure was performed in the case of patients in the respective study group at 21 days from day 5 of admission, in order to capture the same period of time as in the case of patients in the study group. Punch biopsies with a pen with a harvesting diameter of 2 mm were performed. Biopsies were harvested from the edges of the wound.

All skin fragments were fixed in 10% buffered formalin (pH 7.4) for up to 48 h and processed automatically (Excelsior Epredia) in the paraffin incorporation blocks. Then all the slides were stained with Hematoxylin-Eosine (HE) and examined with the Leica DM 3000 LED microscope.

Kits for the preparation of PRP (platelet-enriched plasma) are medical devices that include separator gel tubes based on inert polymer and anticoagulant (sodium citrate 3–4%). Each tube has a precalibrated vacuum for collecting 10/12 mL of blood. The obtaining of platelet-enriched plasma is achieved by centrifuging the blood collected by venopuncture. Centrifugation is carried out for 5 min at 4000 RPM.

After centrifugation, the tube has a transparent yellowish layer in the upper portion, followed by a separating gel barrier and a red lower layer. The tube is removed from the centrifuge, and the plasma is mixed with the platelets on the gel by semi-overturning movements. With the help of the transfer device, the PRP preparation is extracted.

**Figure 1:**
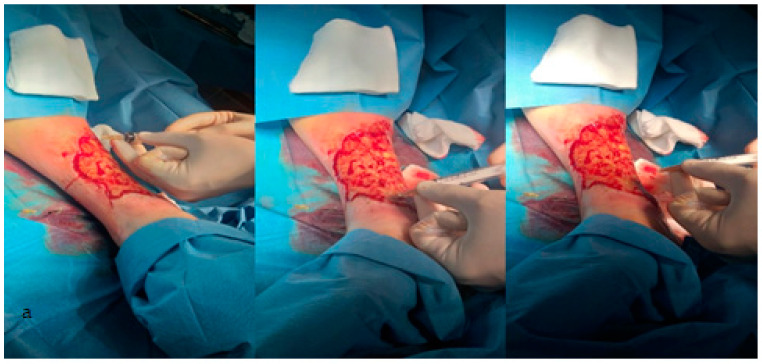

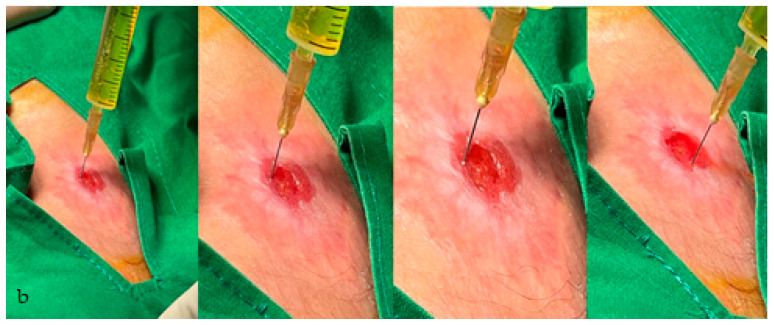
(**a**) Preparation of the patient’s wound bed; (**b**) The treatment—apply platelet-rich plasma.

After preliminary preparation of the patient’s wound bed in the operating room and isolation with sterile fields, the biological material is injected, after 5 days of treatment, obtained by the previously described processing, in the wound edges at a distance of about 2 mm as well as at its level. Without achieving subsequent buffering at the wound level, in order to avoid wiping the biological material, after 10 min, apply a simple sterile dressing, Figure 1a,b. The appearance of the wound is evaluated after 14 days after discharge, both in the control group and in the study group. We mention that our recommendation when discharging patients regarding home treatment was identical in both study batches. It was recommended the toilet with a physiological serum of the wound and the application of a wet compress with an antibacterial solution, the same in all patients included [13].

## 3. Results

After corroborating the data from the questionnaire, we noticed that 76% of the patients in the study group went to the doctor complaining of an atonal wound, and 24% presented themselves complaining of another pathology, who also mentioned the wound at the time of admission. In the control group, 63.3% of the patients attended a consultation for the same main reason as those in the study group. Thus, we noticed that more than half of the patients from both groups had as a reason to present to the doctor the existence of a chronic wound after a previous treatment was performed but mentioned them with a temporary effect.

The background environment is an important factor in the pathologies studied by us, influencing both the appearance of the underlying disease, the complications of the disease and their evolution regarding the treatment.

### Characteristics of the Population

Following the causes of these wounds, within the study group, 25 of the patients were diagnosed with Chronic Obliterating Arteriopathy, 14 of them with complicated type II Diabetes Mellitus, and 11 with Peripheral Venous Disease. Compared to this, in the control group, the main cause of the wounds was also, in this case, Chronic Obliterating Arteriopathy, being present in 11 cases out of the 30 (Table 1).

The dominant symptom of pain in the wound was present at the time of admission in all patients in the study group and in those in the control group. At hospitalization, 40 of the 50 patients were awarded between 8 and 10 points on a scale of 1–10, where 10 represented pain with an impact on mobilization. In the control group, 24 of the patients were given between 8–10 points for the same symptom, which, in this case, was the dominant symptom. Thus, in both groups, there is the same percentage, 80%, in terms of the dominant symptom and the degree of damage to the patients.

**Figure 2:**
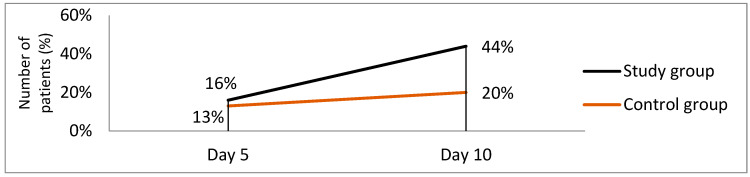
The evolution of the general condition under treatment compared to the two groups.

The pathologies studied by us can, in certain cases, influence the patients’ mobility. We compare the evolution of the mobility scale between the two groups: the initial value in the study group, 14% of the patients gave 5 points on a scale from 0 to 5, where 5 represents the impossibility of walking with the respective limb and 0 shows that walking is not influenced; on day 10, 30% of patients scored 0 points. In the reference group, 13% received 5 points on admission, and 20% received 0 points on the same rating scale (Figure 2).

It is observed that on the 10th day of treatment, the value of the mobility scale between the two groups decreases. The decrease was faster and was maintained until day 10 in the study group, as shown in Table 2. This has been clinically correlated with more rapid improvement in symptoms of the study group.

**Figure 3:**
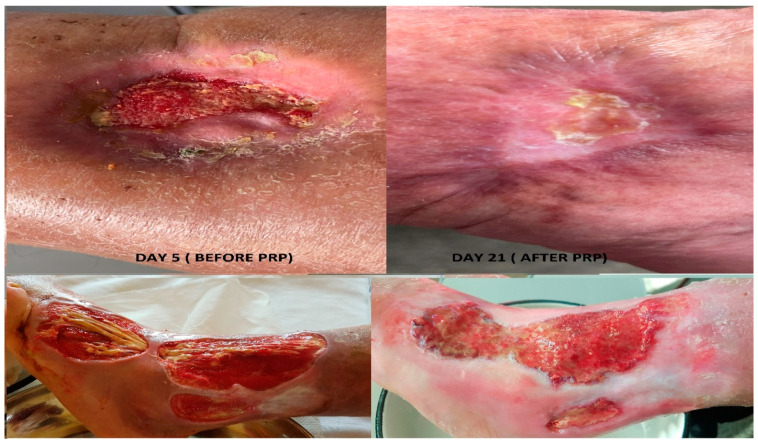
Local evolution of the wound after being injected with platelet-rich plasma.

A promising result was also observed in the evolution of the general condition of the patients. The impact of PRP therapy is obviously a much more positive one compared to patients who have not been injected with platelet-rich plasma. Figure 3 shows the evolution and beneficial effect that this procedure offers to patients and the fact that the treatment was similar in both groups of patients except for PRP therapy in the study group.

The local evolution of the wound was a favorable one regarding the patients who were injected with platelet-rich plasma. The size of the wound has reduced considerably in most patients, Figure 3.

**Figure 4:**
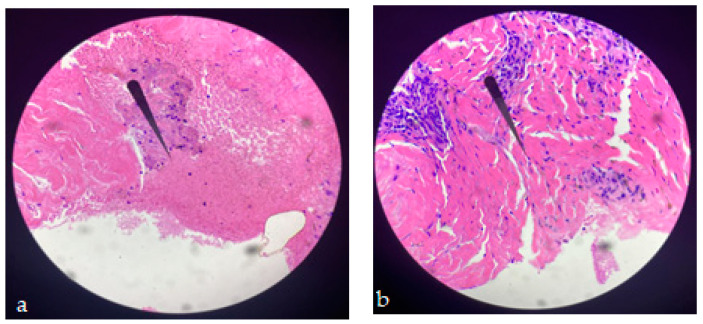
(**a**) The image shows skin ulceration with areas of (superficial) necrosis mixed with inflammatory cells. Epidermis cannot be seen at HE 100× (before; internal biopsy). (**b**) A small area of residual necrosis can be seen, located superficially, and numerous fibroblasts (cells with a cigar-shaped nucleus) are present underneath, which denotes an active regeneration phenomenon. Numerous parallel collagen fibers can still be seen, which will support the healing process. HE 100× (after; biopsy day 21).

We compared histopathological results in both groups of patients as follows: the bioptic sample from admission in the case of both batches of patients, respectively the bioptic sample collected at 21 days from the 5th day of admission, at which time the plasma was injected at the wound level in the study group, to better understand the changes brought by this concentrate. The evolution under this treatment was favorable, accelerating the wound healing phases in most cases. Objectively, the appearance of the wound was improved, given that 54% of the patients in whom this therapy was used came to the control with the wound in the epithelization phase result that was not obtained within the reference group where only a percentage of 6.5% presented themselves at the same stage of healing, Figure 4a,b.

Regarding the study group, 21 days after the application of the PRP injection procedure at the wound level, only 2% of the patients did not show up; 98% of them presented according to the requirements of the study in different phases of wound healing. Thus, 54% of the patients had the wound in the epithelialization stage, 14% in the scarring phase, 22% in the advanced granulation phase, 2% with a wound with stationary evolution, and 4% with unfavorable evolution, the latter requiring re-admission and reassessment of the therapeutic scheme. In the reference group, 46% of the subjects presented the wound with inpatient evolution, 20.5% unfavorable evolution requiring re-admission and reassessment of the therapeutic scheme, of which we mention that 18% involved a superinfection, probably from the non-observance of the hygiene measures at home and the incorrectly applied treatment, 13.5% in the granulation phase, 6.5% in the epithelialization phase, respectively 13.5% did not come to the control, we believe for reasons of presentation in another medical service.

We consider, after collecting the data as well as after the anamnestic and objective examination, that this PRP therapy is beneficial for these patients, stimulating and accelerating the healing process.

## 4. Discussion

Wound care is becoming an increasingly complex clinical practice, especially with the growing need for innovative wound care treatments [14].

Comorbidities often alter wound healing, which leads to an increased risk of developing chronic wounds that do not heal. As such, it is necessary to identify the prevalence of these comorbidities [15]. In our study, patients with these comorbidities, such as valvular insufficiency, various cardiopathies, essential hypertension, and chronic hepatopathies, already had a chronic treatment recorded by specialist doctors. We thus support the idea that these comorbidities are under medicinal control, influencing as little as possible the healing phases.

In a chronic wound, the physiological process of timely repair is disrupted, and the healing time can vary from weeks to years. Many factors (for example, infection, diabetes, cardiovascular diseases, kidney failure, nutritional deficiency, incontinence, immobility, and medication) prevent the healing of wounds [16].

Many active therapies appeared and disappeared from use. Not only must these therapies stimulate healing, but also be cost-effective in the context of the local health system. If active therapy is selected, it is imperative to conduct a consistent and accurate evaluation of the wound so that the progression of the wound in any direction can be determined and the therapy interrupted promptly if the wound is not on a healing line [17].

Removal of devitalized tissue or debris, treatment of inflammation and/or infection, maintaining wound moisture balance, and preparing wound bed edges are the main components of wound management and can serve as new targets for innovation [18,19,20]. As such, the effective use of dressings and wound bed management devices and the start of wound healing is essential to ensure optimal care for ulceration [21].

Chronic wounds often lead to the accumulation of necrotic tissues, which require treatment to facilitate healing. The purpose of debridement of the wound bed is to remove necrotic tissue, reduce pressure, inspect the underlying tissue, eliminate the dead space that houses bacteria, drain pus, and optimize topical preparations in an attempt to stimulate healing. Debridement has long been recognized as necessary for the management of chronic wounds [22] and consists of several methods, including surgical, autolytic, chemical, larval, mechanical, hydro surgery, and ultrasonic methods or a combination of these. Surgical debridement is traditionally perceived as the standard gold standard of debridement; however, no form of debridement is superior to another, and there is insufficient data from randomized trials controlled in surgical wounds, venous foot ulcers and diabetic foot ulcers on which to base current practice [23,24,25]. In our study, we decided to carry out surgical and chemical debridement where appropriate, as well as local curettage to stimulate circulation and local granulation. We decided to perform a 5-day treatment before the injection of the rich plasma into platelets precisely from the principle of making a bed as clean as possible of the wound and for a careful and correct preparation performed for the reason of obtaining a moment as adequate as possible to instill the substance biology of PRP.

Evaluation of the edges of the wound may indicate the progress of contraction and epithelization of the wound and confirm if the current treatment of the wound is efficient. A 20–40% reduction in wound surface area after 2 and 4 weeks of treatment proved to be a reliable predictor of healing [26]. It is also important to assess the condition of the surrounding skin since dry or macerated edges can prevent healing.

Stem cells have been theorized to help promote wound healing by migrating over the wound bed and secreting chemokines and growth factors to induce angiogenesis and extracellular matrix remodeling [27]. Autologous plasma gel rich in platelets consists of cytokines, growth factors, and a fibrin scaffold derived from the patient’s blood. A recent systematic review showed that this treatment conferred a certain increase in the rate of wound healing compared to a placebo gel or standard care [28].

Wound healing processes are regulated by a complex of growth factors and cytokines released by platelet granules. Therefore, platelets (PLT) not only prevent blood loss but also promote tissue regeneration, improve collagen synthesis, and trigger angiogenesis and immune response by releasing growth factors and cytokines [29,30,31].

Using platelets as a regenerative agent in wound healing/tissue regeneration by instant preparation without requiring advanced preparation facilities is an advantage. They are safe and natural. Due to their direct extraction from the patient’s blood, they are natural, and there is no possibility of a negative immune response and blood contamination [32].

The effectiveness of PRP-based therapies through its methods of preparation, manipulation, classification and application in wound healing is proven to be a regenerative therapy with a beneficial effect. PRP has an important role in hemostasis, angiogenesis, stem cell migration, innate immunity, proliferation and wound healing [32]. The development of new wound healing therapies to improve skin wound healing is a current requirement. Regenerative therapies are becoming more and more used since they are invasive procedures. Platelet-rich plasma (PRP) has been shown to be very good in its potential to stimulate and accelerate the healing process of wounds. The cytokines and growth factors that form PRP play a major role in the healing process. PRP represents a safe and cost-effective treatment in the healing process of skin wounds by reducing the recovery period, thus improving the quality of life of patients [33,34].

Platelet-rich plasma (PRP) consists of autologous plasma with a platelet concentration five times higher than the basal level due to an extraction and concentration process (described above). The use of concentrated plasma for wound healing processes has been explored over the past decade. Most of these products are often improperly referred to as PRP, such as the concentrations of the original transfusion platelets, which make it difficult to differentiate between the different protocols [35].

Several studies on the clinical use of PRP have already been conducted. Most of the literature ranges from dental medicine to maxillofacial surgery, from dermatology and aesthetic medicine to orthopedics and sports medicine and even neurology [36,37,38,39,40,41]. PRP not only increases the rate of wound healing but also reduces neurological and neuropathic pain associated with injuries. The use of autologous platelet concentrates accelerates healing in dental implant surgery, orthopedic surgery, muscle and tendon repair, skin ulcers, and orifice repair in eye surgery and cardiac surgery. All these therapeutic properties are possible because PLT blood products are rich in growth factors and cytokines that stimulate and accelerate the wound-healing process. Although there has already been a lot of research, there is still a lot of work to be done. For example, the absence of a standardized protocol for the preparation of PRPs and the lack of universally accepted terminology makes it difficult to compare studies and their results. Moreover, the available studies remain controversial about the real benefit, especially in the treatment of ulcers, and there is no agreement on the duration of therapy because FMPs are released quickly and require more applications to maintain their therapeutic effect. Finally, there is no information about therapeutic doses of PRP and the possible interference of some drugs on the release of PGF16 [41,42,43,44].

Evaluating the international library of publications and highlighting the usefulness of platelet-rich plasma, we decided to conduct this study on several patients with wound beds already prepared for injection and the evaluation of structural changes from a histopathological point of view as well as objectives in terms of local evolution and the general condition of the patients. The usefulness of this product on a large scale in various medical fields, including dermatology, neurology, orthopedics, dentistry, and aesthetics, has laid the basis for our study on the use of plasma concentrations in the healing of chronic wounds from different pathologies.

From the point of view of the applicability of home treatments, taking into account that certain patients tried various techniques before presenting in the medical field [45], we noticed that those who used more than two local topicals or more than two topicals in combination with various antiseptic substances had a more serious evolution compared to those who used less than two topical or alternative topical/antiseptic treatment, they have a much better evolution and response to laser therapy as well.

We chose the approach of this topic of study due to the fact that the presence of associated pathologies that lead to the appearance and maintenance of chronic ulcers are more and more numerous in conjunction with the avoidance of early presentation of patients to the doctor [44]. The effect is a negative one on the healing phases, given that it further delays the stages in this process.

A percentage of 2% with the wound with stationary evolution and 4% with unfavorable evolution, the latter requiring re-admission and reassessment of the therapeutic scheme, the medical problem not being solved or even aggravated. Based on this observation, we will aim to conduct a future study in which we will analyze other medical methods by which, even in combination with various more serious or present comorbidities for a long time, it could lead to an accelerated cure in combination with the PRP procedure.

## 5. Conclusions

The local evolution of the wound was a favorable one regarding the patients who were injected with platelet-rich plasma, and the size of the wound has reduced considerably in most patients. A promising result was also observed in the evolution of the general condition of the patients. The impact of PRP therapy is a much more positive one compared to patients who have not been injected with platelet-rich plasma.

## Figures and Tables

**Table 1:** Patient demographics and disease characteristics at baseline visit.

	Study Group *n* = 50 (100%)	Control Group *n* = 30 (100%)
Demographic characteristics		
Age in years, mean	68.24	60.2
Men	14 (26.92%)	12 (63.33%)
Women	36 (73.07%)	11 (36.66%)
Rural	11 (22%)	13 (43.33%)
Urban	39 (78)	17 (56.66%)
Wound etiology, *n* (%)		
Peripheral Venous Disease	11 (22%)	7 (23.33%)
II Diabetes Mellitus	14 (28%)	2 (6.66%)
Chronic Obliterating Arteriopathy	25 (50%)	11 (36.66%)

**Table 2:** Comparison of scale mobility on Day 5 and Day 10.

	Study Group MD ± DS	Control Group MD ± DS	*p* Value
Day 5	1.85 ± 1.66	0.43 ± 0.59	<0.001
Day 10	4.64 ± 0.49	1.21 ± 0.83	0.01
P^Study-Control^	0.003	0.342	<0.001

## Data Availability

Not applicable.
